# Skeletal muscle mass obtained by anthropometric equation and presence of sarcopenia in postmenopausal women

**DOI:** 10.61622/rbgo/2024AO09

**Published:** 2024-03-15

**Authors:** Thaís Loureiro Felipe, Patrícia Paula da Fonseca Grili, Camila Vilarinho Vidigal, Ben-Hur Albergaria, Geise Ferreira da Cruz, José Luiz Marques-Rocha, Valdete Regina Guandalini

**Affiliations:** 1 Universidade Federal do Espírito Santo Vitória ES Brazil Universidade Federal do Espírito Santo, Vitória, ES, Brazil.

**Keywords:** Muscle mass, Sarcopenia, Anthropometry, Postmenopause, Overweight, Obesity, Body composition, Bioelectrical impedance, Skeletal Muscle

## Abstract

**Objective:**

To analyze the amount of muscle and the presence of sarcopenia in postmenopausal women using different methods, verifying the agreement between them as to skeletal muscle mass (SMM).

**Methods:**

This cross-sectional observational study was conducted with postmenopausal women aged ≥ 50 years. SMM was obtained from a predictive equation, Bioelectrical Impedance (BIA), and Dual Energy X-Ray Absorptiometry (DXA). The skeletal muscle mass index (SMI) and the appendicular skeletal muscle mass index (ASMI) were calculated. The cut-off point of SMI was determined for the population itself. The agreement between the SMI obtained using the different methods was verified. Sarcopenia was diagnosed according to the criteria proposed by the European Working Group on Sarcopenia in Older People 2 (EWGSOP2). The significance level adopted for all tests was 5.0%.

**Results:**

A total of 112 women were evaluated, with an average age of 66.1 ± 5.65 years. Among them, 51.8% were sufficiently active and 43.8% were overweight and obese. The SMI cut-offs were 6.46 kg/m^2^ for the predictive equation and 7.66 kg/m^2^ for BIA, with high sensitivity and specificity. There was an excellent agreement in the identification of SMM by the predictive equation (0.89 [0.824-0.917], p < 0.001) and BIA (0.92 [0.883-0.945], p < 0.001), in reference to DXA. The prevalence of sarcopenia was 0.9%, 1.8%, and 2.7% according to BIA, DXA, and the predictive equation, respectively.

**Conclusion:**

The predictive equation showed the expected agreement in estimating skeletal muscle mass in postmenopausal women, offering a viable and accurate alternative.

## Introduction

Sarcopenia is a musculoskeletal disease associated with the aging process, with a consequent reduction in functional capacity, physical disability, and worsening of quality of life, in addition to being associated with falls, fractures, hospitalization, institutionalization, morbidities, and mortality.^(1,2)^

The European Working Group on Sarcopenia in Older People 2 (EWGSOP2) defined the stages of the disease according to criteria that include low muscle strength, low muscle quantity and quality, and low physical performance.^(3)^ Considering that skeletal muscle mass (SMM) is the main parameter used to confirm sarcopenia, different methods have been proposed to identify it, including Dual Energy X-Ray Absorptiometry (DXA), Bioelectrical impedance (BIA), Magnetic Resonance Imaging (MRI), and Computed Tomography (CT).^(3)^

All the aforementioned methods of measuring muscle mass (MM) are indirect, lacking precise accuracy, and each has advantages and disadvantages^(4)^ DXA is considered the reference standard due to its less invasive nature, low radiation emission, and greater accessibility when compared to CT or MRI, although it does not distinguish between muscle fat infiltration and is unable to measure trunk SMM.^(5)^ CT, on the other hand, evaluates muscle attenuation, intra and intermuscular fat infiltration, and is highly reproducible, but it has a high cost, low accessibility, convenience, and high radiation test. Finally, MRI also has high reproducibility, evaluates extra and intramyocellular fat, and emits low radiation, but it costs even more and is less accessible than CT. Both are considered gold standard methods. Although MRI is the best for MM assessment, its use has been restricted to research only.^(6)^

Although the recommended exams are highly precise in the measurement of SMM, their use as a routine procedure is limited. Thus, low-cost, easily applicable, and non-invasive screening and identification methods that can diagnose sarcopenia early and rapidly are needed.^(7,8)^

The predictive equation proposed by Lee et al.^(8)^ estimates SMM from anthropometric and sociodemographic variables. As it is easy to apply in clinical practice, does not require expensive equipment, and provides immediate results, it can become an efficient tool for identifying SMM, having been used in previous studies as an alternative in the diagnosis of sarcopenia.^(7)^

Esteves et al.^(7)^ used this equation to identify SMM and determine specific cut-off points for the diagnosis of sarcopenia in elderly people living in the northern region of Brazil. The results showed that sarcopenic elderly people had lower mean values of anthropometric measurements when compared with non-sarcopenic individuals, suggesting that these measurements may be indicators of muscle impairment and thus used for sarcopenia screening.^(7)^

Faced with the need to expand and facilitate the diagnosis of sarcopenia, associated with the scarcity of studies that analyze other methods of identifying this disorder in postmenopausal women, this study aimed to: (1) identify the amount of muscle and the presence of sarcopenia using different methods and (2) verify the agreement between them as to SMM.

## Methods

This observational cross-sectional study evaluated 140 postmenopausal women aged ≥ 50 years cared for at the general gynecology and obstetrics outpatient clinic of a Brazilian university hospital, from June 2019 to March 2020. As these are secondary analyses of a previous study, information on sample calculation and sample selection has been previously published.^(9)^ For this study, women who had been menopausal for at least 12 months were included and those using hormone replacement therapy (HRT) and who did not present a DXA report were excluded.

From a semi-structured questionnaire, sociodemographic information was collected, such as self-reported color^(10)^ (black, brown, white), age (years), educational level (no schooling, elementary school, high school, higher education), marital status (with a partner, without a partner), and employment status (with and without employment). Lifestyle factors such as smoking (smoker, non-smoker), alcohol consumption (consumes, does not consume), and physical activity level (PA) were obtained by the International Physical Activity Questionnaire (IPAQ), long version,^(11)^ considering only the sum of issues related to leisure and transport to avoid overestimation. Women who reported performing 150 minutes or more of PA per week were classified as "sufficiently active" while those who did not reach the recommendation of the World Health Organization (WHO) were classified as "insufficiently active".^(12)^ As for clinical data, we examined the time since menopause in years and categorized it into "≤ 19 years" and "> 19 years".

To assess nutritional status, body mass (kg) and height (m) were measured according to recommended techniques.^(13)^ The body mass index (BMI) was calculated by dividing the mass by the squared height (kg/m²) and classified according to age. For women under 60 years of age, we used the cut-off points proposed by the WHO,^(14)^ while for older women, the reference standard proposed by the Pan American Health Organization (OPAS)^(15)^ was used.

Skeletal muscle mass was obtained by three different methods: a predictive equation, BIA, and DXA. The equation proposed by Lee et al.,^(8)^ validated for use in elderly Brazilians^(16)^ and used in previous population-based studies^(17,18)^ to determine SMM, reads as follows: SMM (kg) = (0.244 x body weight) + (7.8 x height in meters) – (0.098 x age) + (6.6 x sex) + (race – 3.3)

This equation considers the parameters body mass, height, sex, age, and race. For the sex variable, 0 = female and 1 = male; for race, 0 = white and indigenous, - 1.2 = brown, and 1.4 = black and brown.^(8)^

To obtain SMM by BIA, we used the InBody® 230 model. Participants were instructed on the protocol to be followed before the exam. The skeletal muscle mass index (SMI) was calculated by dividing SMM – obtained through BIA and the Lee et al.^(8)^ equation – by squared height (kg/m²). The cut-off points of each method were determined for the study population itself.

Appendicular skeletal muscle mass (ASM) was obtained from the whole-body DXA, performed using the GE Lunar Prodigy Advance® device and the GE Encore® software, version 14.10, configured to use the National Health and Nutrition Examination Survey (NHANES) reference database. The appendicular muscle mass index (ASMI) was calculated by the ratio between ASM (kg) obtained by DXA and height (m) squared. ASMI values < 5.5 kg/m² were taken as an indication of low muscle quantity.^(3)^

The participants were instructed to fast for 4 hours, empty the bladder up to 30 minutes before the exam, not perform physical activity 12 hours before the exam, and remove metallic objects, in addition to remaining immobile and silent during the exam. The procedure took place in an acclimatized room with temperatures between 20 and 25 °C. To minimize inter-observer variation, all densitometry exams were performed by a certified radiology technician trained for this type of exam and interpreted and reported by a single specialist physician.

For the diagnosis of sarcopenia, the criteria recommended by the EWGSOP2^(3)^ were used: Handgrip Strength (HGS), SMI, and the Timed Up and Go (TUG) test.

Three stages of sarcopenia were considered: (1) Probable Sarcopenia, characterized by reduced HGS; (2) Sarcopenia, confirmed by the presence of low muscle quantity, identified via the three SMM assessment methods employed here; and (3) Severe Sarcopenia, confirmed when muscle strength, muscle quantity/quality, and physical performance (according to the TUG test) were below the recommended cut-off points.^(3)^

To assess HGS, a Jamar^®^ manual dynamometer was used. The test was performed using the method recommended by the American Association of Hand Therapy (ASHT).^(19)^ The participant remained seated, with the spine erect, knees flexed at 90°, the shoulder positioned in adduction, the forearm supported, and the elbow flexed at 90°. The procedure was performed three times on the dominant hand (DHGS) and three times on the non-dominant hand (NDHGS), with maximum effort for about 5 seconds and a 1-minute interval between measurements.^(19)^ The test was not performed on participants who underwent hand, arm, or forearm surgery less than 60 days prior to the assessment.^(19)^ The cut-off points < 16.0 kg for women, defined by the European Consensus on Sarcopenia were considered.^(3)^ Physical performance was assessed by the TUG test, in which the individual gets up from a chair without supporting the arms or assistance and walks for 3 meters, returning to the starting point and sitting down again.^(20)^ The entire process was timed by the researcher and repeated three times. A walking time of ≥ 20 seconds was considered inappropriate.^(3)^

The sample was characterized based on frequency distribution and estimation of measures of central tendency and dispersion. The normality of the study variables was assessed using the Kolmogorov-Smirnov test.

Receiver Operating Characteristics (ROC) curves were constructed to determine the SMMI cut-off points obtained by BIA and the predictive equation, as sarcopenia discriminators. The area under the ROC curve (AUC), sensitivity, and specificity were also determined with 95% CI. The Kappa coefficient was calculated to verify the agreement between the different methods for calculating SMM and SMI, taking as references the cut-off points proposed by the EWGSOP2. We used the categories proposed by Landis and Koch,^(21)^ according to the degree of agreement found: < 0, no agreement; 0-0.19, poor agreement; 0.20-0.39, slight agreement; 0.40-0.59, moderate agreement; 0.60-0.79, substantive agreement; and 0.80-1.00, almost perfect agreement. The Intraclass Correlation Coefficient (ICC) was calculated to verify agreement between SMM measurements and classified according to Cicchetti's^(22)^ second degree of correlation in: < 4.0, weak; 0.4-0.59, fair; 0.60-0.74, good; 0.75-1.0, excellent.

The analyses were carried out using the Social Package Statistical Science (SPSS)^®^ for Windows version 22.0 program, and the significance level adopted for all tests was 5.0%.

Participation was voluntary and consent was given in writing by signing the Free and Informed Consent Term, in accordance with Resolution CNS 466/12 of the Ministry of Health.^(23)^ The project was approved by the Research Ethics Committee of the Federal University of Espírito Santo, under protocol number 2,621,794.

## Results

The final study sample consisted of 112 postmenopausal women. There was a predominance of women who declared themselves brown (55.4%), with elementary education (59.9%), with a partner (52.7%), employed (83.0%), and with time since menopause ≤19 years (50.9%). As for lifestyle habits, 51.8% were classified as sufficiently active, 87.5% did not consume alcohol, and 95.5% did not smoke. Regarding nutritional status, 43.8% were classified as overweight according to BMI (Table 1).

**Table 1:** Sociodemographic, lifestyle, and nutritional status of postmenopausal women

Variables		n(%)
Age, years (SD)		66.1 ± 5.65
Age groups		
	50-59.0	14(12.5)
	≥ 60.0	98(87.5)
Color		
	Brown	62(55.4)
	White	41(36.6)
	Black	9(8.0)
Educational level		
	No schooling	10(8.9)
	Elementary school	67(59.9)
	High school	24(21.4)
	University education	11(9.8)
Marital status		
	No partner	53(47.3)
	With partner	59(52.7)
Employment status		
	Employed	93(83.0)
	Unemployed	19(17.0)
Physical activity level		
	Sufficiently active	58(51.8)
	Insufficiently active	54(48.2)
Alcohol Consumption		
	Does not consume	98(87.5)
	Consumes	14(12.5)
Smoking		
	Non-smoker	107(95.5)
	Smoker	5(4.5)
Time since menopause (years)		
	≤ 19	57(50.9)
	> 19	55(49.1)
Nutritional status		
	Underweight	18(16.1)
	Normal weight	45(40.2)
	Overweight	18(16.1)
	Obesity	31(27.6)

**Figure 1:**
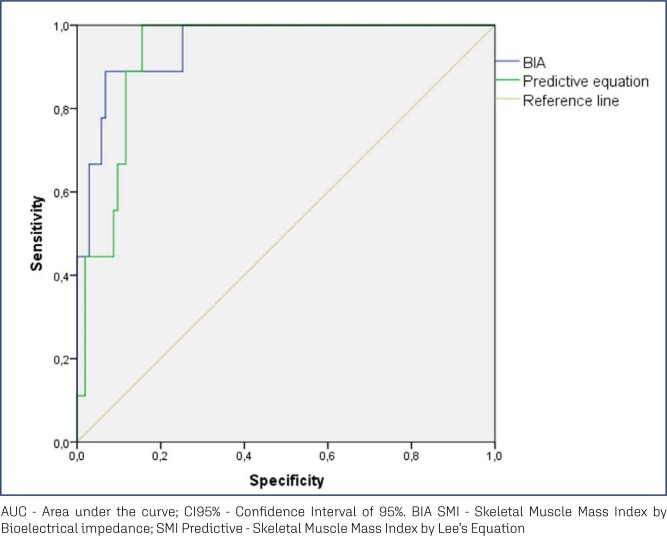
Areas under the ROC curve to discriminate between the skeletal muscle mass index calculated from the predictive equation and by bioelectrical impedance analysis in postmenopausal women

The results of the area under the ROC curve indicated coefficients greater than 0.7, which were considered acceptable (Figure 1). The cut-off points of the total study population for SMI derived from the predictive equation and BIA were 6.46 kg/m² and 7.66 kg/m², with high sensitivity (100% and 89%) and specificity (84.5% and 93%), respectively.

**Figure 2:**
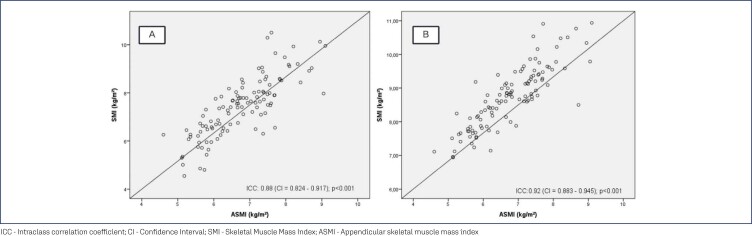
Intraclass correlation coefficient (ICC) between the ASMI obtained from DXA and the SMI obtained by the predictive equation (A) and by the bioelectrical impedance (B) in postmenopausal women

Table 2 shows the correlation between the ASMI assessed by DXA and the SMI obtained by the predictive equation and BIA in postmenopausal women. SMI values obtained through the predictive equation (0.88 [0.824-0.917], p < 0.001) (Figure 2A) and BIA (0.92 [0.883-0.945], p < 0.001) (Figure 2B) showed significant ICCs when compared with the ASMI determined by the DXA.

**Table 2:** Correlaciona between the Skeletal Muscle Index Predicitive and Eletrical Bioimpedance

	Sensitivity (%)	Specificity (%)	AUC (CI95%)	Cut-off	p-value
SMI Predictive	100.0	84.5	0.930 (0.878 – 0.981)	6.46	< 0.001
SMI BIA	89.0	93.0	0.951 (0.896 – 1.00)	7.66	< 0.001

AUC - Area under the curve; CI - Confidence Interval of 95%; SMI - Skeletal Muscle Index; Predictive - Lee's equation; BIA - Bioelectrical impedance.

When analyzing the distribution of the categories HGS and TUGT according to skeletal muscle mass indexes obtained by different methods, we did not observe significant differences between groups (Table 3).

**Table 3:** Distribution of handgrip strength and Timed Up-and-Go test according to skeletal muscle mass indexes obtained by different methods

Variables		ASMI (DXA) n(%)		SMI (Predictive) n(%)		SMI (BIA) n(%)	
		Adequate	Reduced	Adequate	Reduced	Adequate	Reduced
		103(92.0)	9(8.0)	87(77.7)	25(22.3)	98(87.5)	14(12.5)
HGS							
	Adequate	98.0(93.3)	7(8.7)	83.0(79.0)	22(21.0)	92(87.6)	13 (12.4)
	Reduced	5(71.4)	2(28.6)	4(57.1)	3(42.9)	6(85.7)	1 (14.3)
	*p-value*	0.098		0.184		1.000	
TUGT							
	Adequate	100(91.7)	9(8.3)	84(77.1)	25(22.9)	95(87.2)	14(12.8)
	Reduced	3(100.0)	-(-)	3(100.0)	-(-)	3(100.0)	-(-)
	p-value	1.000		1.000		1.000	

Fisher's exact test; HGS - Handgrip Strength; ASMI - Appendicular Skeletal Muscle Mass Index; SMI - Skeletal Muscle Mass Index; DXA - Dual Energy X-Ray Absorptiometry; BIA - Bioelectrical impedance; Predictive - Lee's equation; TUGT - Timed Up-and-Go Test

Among the women studied, 6.3% exhibited probable sarcopenia. The prevalence of sarcopenia, as determined by different methods, was 0.9% using BIA, 1.8% with DXA, and 2.7% through the predictive equation (Table 4). None of these methods detected the presence of severe sarcopenia (data not shown).

**Table 4:** Classification of sarcopenia stages according to diagnostic criteria in postmenopausal women

Variables		n(%)
Probable sarcopenia		
	Absent	105(93.8)
	Present	7(6.3)
Sarcopenia (DXA)		
	Absent	110(98,2)
	Present	2(1.8)
Sarcopenia (Predictive)		
	Absent	109(97.3)
	Present	3(2.7)
Sarcopenia (BIA)		
	Absent	111(99.1)
	Present	1(0.9)

DXA - Dual energy X-ray absorptiometry; BIA - Bioelectrical Impedance; Predictive - Lee's equation

## Discussion

The present results showed that the predictive equation used as an alternative to obtain SMI succeeded in identifying sarcopenia in postmenopausal women compared with the reference standard (DXA), considering the cut-off point of the population itself. The prevalence of sarcopenia varied between 0.9% and 2.7% depending on the method used to evaluate SMM.

The prevalence of sarcopenia varies among Brazilian regions. This divergence may be related to the methods and cut-off points used, and to the regional, ethnic-racial, and lifestyle characteristics of the population studied.^(24)^ Diz et al.,^(2)^ in a meta-analysis including 31 randomly chosen studies, investigated the prevalence of sarcopenia in Brazilian elderly individuals aged ≥ 60 years. When considering the EWGSOP^(25)^ criteria, the prevalence of sarcopenia found was 16.0%. When using only muscle mass from the DXA exam and Baumgartner's et al. criteria,^(26)^ the prevalence was 17.0%. When considering only elderly women, the average prevalence in all studies increased to 20%.^(2)^

This high prevalence can be explained by the methods and cut-off points used to identify the disease, which were based on those proposed by the EWGSOP and Baumgartner et al.,^(25,26)^ in addition to the other sarcopenia phenotypes. On the other hand, our study used as a basis, in addition to the EWGSOP2 cut-off points,^(3)^ those developed specifically for the population studied.

On the other hand, Mazocco et al.,^(27)^ when evaluating the prevalence of sarcopenia in elderly women living in urban and non-urban areas of Brazil's southern, observed that 2.4% of this population had sarcopenia when using EWGSOP (2010).^(27)^ This result corroborates those found in the present study. This similarity may be related to similar characteristics such as age group, level of physical activity, non-smoking habit, and overweight, in addition to all women being in the post menopause period and not using HRT.

As for continuous SMI values, both the ones obtained by the predictive equation and those by BIA showed excellent agreement in reference to ASMI measured by DXA, both for the total population of the study and for elderly women. However, when determining the SMI cut-off points by the ROC curve, we observed that the SMI derived from BIA was not able to diagnose sarcopenia in the group evaluated, which reinforces the need for care in the choice of diagnostic method, in the application of the pre-established cut-off points, and in the interpretation of results, in order to prevent sarcopenic individuals from not being identified.

Previous national studies^(7,17,18)^ showed similar cut-off points, which raises the hypothesis that the ASMI cut-off point defined by the EWGSOP2 may not fully meet the Brazilian population, since it is based on different peoples from different countries, especially indigenous, African, and European immigrants.^(28)^

Fernandes et al.^(24)^ developed cut-off points according to the 20th percentile to screen for sarcopenia in elderly people living in the northeast region of the country and compared it to the values recommended by the EWGSOP2, Foundation for the National Institutes of Health (FNIH), and the International Working Group on Sarcopenia (IWGS). The cut-off point for the SMI of women in this population was 5.52 kg/m², a value similar to that from the EWGSOP2.^(24)^ By analyzing the prevalence of sarcopenia in women, the results obtained were 4.6% by the EWGSOP2, 11.7% by the FNIH, and 27.5% by the IWGS. When the cut-off points determined by the 20th percentile were used, prevalence numbers of 4.6%, 4.7%, and 5.1% were observed, respectively,^(24)^ the latter being consistent with our results.

Liu et al.,^(29)^ when comparing different diagnostic criteria for sarcopenia in participants aged 50 years and over, showed that the prevalence of sarcopenia ranged from 8.2% to 57.4%.^(29)^ This result attests to the great variation that can occur due to the different cut-off points and consensus used, which reinforces the importance of either developing specific cut-off points for each population or applying and interpreting the ones available in a judicious way.

Assessment of muscle mass is an essential step in the diagnosis of sarcopenia. DXA is considered the standard reference method to determine muscle quantity.^(3)^ Despite being highly precise, its use as a routine procedure becomes unfeasible due to the high cost, preparation, time spent to perform it, the need for trained personnel and specific software for its performance, and availability in clinical practice.^(30)^

The predictive equation proposed by Lee et al.^(8)^ estimates total skeletal muscle mass, allowing the diagnosis of sarcopenia quickly and efficiently because of its high specificity and sensitivity, and it can be used on a large scale and in clinical practice to discriminate women with this condition. The anthropometric and sociodemographic variables used in the equation can be obtained using low-cost, non-invasive, and unsophisticated instruments, facilitating access to public health services.

Although BIA is a diagnostic method recommended by the EWGSOP2, it failed in identifying sarcopenia in the women evaluated here. This can be explained by the fact that the BIA equipment measures muscle mass indirectly, providing an estimate based on the electrical conductivity of the entire body.^(29)^ In addition, BIA algorithms are derived and incorporated by the manufacturer based on a specific population and vary according to the device used, so when applied to populations other than the validated sample, SMM can be overestimated, as seen in other studies.^(28,31)^ That was indeed demonstrated by the cut-off point determined in the present study, which is higher than that of the EWGSOP2 and the predictive equation.

Although our results are encouraging, it is important to acknowledge that this study has some limitations. Participants aged 50 years or older were included, but the cut-off points recommended in the guidelines are not specific for this age group, which may have influenced the prevalence found. The sample was restricted to postmenopausal women and came from a single location, which may limit the generalization of results to other population groups. In addition, the use of DXA as a reference standard, although widely accepted, may also present some limitations regarding the accurate assessment of SMM.

Due to the various consequences of sarcopenia and the significant annual increase in the elderly population in Brazil and worldwide, the early and rapid identification of sarcopenia, as well as its prevention, can reduce the costs of future treatment of aggravated cases of the disease and its consequences at the level of public health.^(32)^

The results of this study have significant clinical implications, as early identification of sarcopenia in postmenopausal women is critical for implementing appropriate interventions. The adoption of a predictive equation as a screening tool, as proposed by Lee et al.,^(8)^ can facilitate the regular monitoring of changes in skeletal muscle mass, allowing early interventions and potentially improving quality of life and preventing complications related to muscle loss. However, further research is still needed to validate the effectiveness of this equation in different population groups and to investigate possible modifications over time.

## Conclusion

This study demonstrated that assessing skeletal muscle mass through the predictive equation in postmenopausal women is a viable and accurate alternative. The use of the specific cutoff point for our population enabled the efficient identification of sarcopenia in comparison with the reference standard. These results are promising for diagnosing and monitoring sarcopenia in this population group, providing a more accessible and less expensive approach to assessing muscle health.
